# A Genetic Association Test Accounting for Skewed X-Inactivation With Application to Biotherapy Immunogenicity in Patients With Autoimmune Diseases

**DOI:** 10.3389/fmed.2022.856917

**Published:** 2022-06-01

**Authors:** Signe Hässler, Sophie Camilleri-Broët, Matthieu Allez, Florian Deisenhammer, Anna Fogdell-Hahn, Xavier Mariette, Marc Pallardy, Philippe Broët

**Affiliations:** ^1^INSERM UMR 959, Immunology-Immunopathology-Immunotherapy (i3), Sorbonne Université, Paris, France; ^2^Assistance Publique Hôpitaux de Paris, Hôpital Pitié Salpêtrière, Biotherapy (CIC-BTi), Paris, France; ^3^OPTILAB-MUHC, Division of Pathology, Department of Laboratory Medicine, McGill University Health Center, Montreal, QC, Canada; ^4^Department of Gastroenterology, Hôpital Saint-Louis, AP-HP, Université Paris-Diderot, Paris, France; ^5^Department of Neurology, Medical University of Innsbruck, Innsbruck, Austria; ^6^Department of Clinical Neuroscience, Karolinska Institutet, Stockholm, Sweden; ^7^Université Paris-Saclay, INSERM UMR1184, Center for Immunology of Viral Infections and Autoimmune Diseases, Assistance Publique - Hôpitaux de Paris, Le Kremlin Bicêtre, France; ^8^Université Paris-Saclay, INSERM, Inflammation, Microbiome, Immunosurveillance, Châtenay-Malabry, France; ^9^University Paris-Saclay, CESP, INSERM, AP-HP, Université Paris-Sud, Hôpitaux Universitaires Paris-Sud, Villejuif, France

**Keywords:** immunogenicity, anti-drug antibodies, biotherapy, autoimmune disease, X-chromosome, skewed X-Chromosome inactivation, additive hazard model

## Abstract

Despite being assayed on commercialized DNA chips, the X chromosome is commonly excluded from genome-wide association studies (GWAS). One of the reasons is the complexity to analyze the data taking into account the X-chromosome inactivation (XCI) process in women and in particular the XCI process with a potentially skewed pattern. This is the case when investigating the role of X-linked genetic variants in the occurrence of anti-drug antibodies (ADAs) in patients with autoimmune diseases treated by biotherapies. In this context, we propose a novel test statistic for selecting loci of interest harbored by the X chromosome that are associated with time-to-event data taking into account skewed X-inactivation (XCI-S). The proposed statistic relies on a semi-parametric additive hazard model and is straightforward to implement. Results from the simulation study show that the test provides higher power gains than the score tests from the Cox model (under XCI process or its escape) and the Xu et al.'s XCI-S likelihood ratio test. We applied the test to the data from the real-world observational multicohort study set-up by the IMI-funded ABIRISK consortium for identifying X chromosome susceptibility loci for drug immunogenicity in patients with autoimmune diseases treated by biotherapies. The test allowed us to select two single nucleotide polymorphisms (SNPs) with high linkage disequilibrium (rs5991366 and rs5991394) located in the cytoband Xp22.2 that would have been overlooked by the Cox score tests and the Xu et al.'s XCI-S likelihood ratio test. Both SNPs showed a similar protective effect for drug immunogenicity without any occurrence of ADA positivity for the homozygous females and hemizygous males for the alternative allele. To our knowledge, this is the first study to investigate the association between X chromosome loci and the occurrence of anti-drug antibodies. We think that more X-Chromosome GWAS should be performed and that the test is well-suited for identifying X-Chromosome SNPs, while taking into account all patterns of the skewed X-Chromosome inactivation process.

## 1. Introduction

Despite the widespread recognition that genes play a role in many complex diseases, it is puzzling that one of the most important biological characteristics, the sex which is determined by the sex chromosomes, is often overlooked in genome-wide association studies (GWAS) (1–3). In practice, most of the GWAS discard this information whereas commercialized genotyping chips include thousands of Single Nucleotide Polymorphisms (SNPs) on the X chromosome. Even for autoimmune diseases that show strong sex differences in prevalence, the analyses are often restricted to the autosomes, thus neglecting X-linked information. Some potential reasons explaining this lack of interest for X chromosomes as compared to autosomes include lower coverage of chromosome X, technical issues regarding genotype calling and imputation and non-standard statistical analyses (4). In the latter case, the methodological problem is due to the fact that the statistical methods should take into account the X-chromosome inactivation (XCI) process on female X-chromosome loci (5, 6).

The main feature that makes the X chromosome different from the autosomal chromosomes is obviously the fact that, except for the pseudo-autosomal regions resulting from the divergence of evolution of sex chromosomes (X and Y), women have two copies while men have only one copy of each gene. This dosage imbalance is in part compensated by inactivation of one X chromosome in females through XCI. In each female cell, one copy of the X chromosome is inactivated. X-chromosome inactivation occurs at random (paternal or maternal), very early in embryonic life and is inherited by all daughter cells through mitosis (5). Females are mosaic, each cell having either the paternal or maternal X-chromosome inactivated. Such mosaic states can also be imposed in the case of gonosome aneuploidies. While the random inactivation process results in roughly a symmetrical (50:50) distribution in most females, skewing of X chromosome inactivation (XCI-S) is observed in some women, leading to a majority of either paternal or maternal X-chromosome inactivation. This skewing might be due either to selective pressure (negative selection) or a stochastic process (random selection in an embryonic stage where a limited number of cells give rise to the different tissues). Moreover, some genes may escape X-chromosome inactivation and remain biallelically expressed (XCE) (7).

In recent years, biopharmaceutical products (BPs) such as therapeutic monoclonal antibodies have become increasingly used in clinical practice and have led to a critical step forward in the treatment of many severe auto-immune diseases. However, for some patients these drugs activate the immune system, leading to the formation of Anti-Drug Antibodies (ADA). The mechanisms leading to drug immunogenicity can either be patient-related (genetic background, immunological status, prior exposure, prior disease) or treatment-related (drug characteristics and formulations, route, dose, frequency of administration) (8, 9). While some genomewide studies have investigated the genetic factors associated with the immunogenic potential of biotherapies (10), to our knowledge, none has investigated the X-chromosome.

To analyze the X chromosome in association studies, several test statistics have been proposed and implemented for case-control studies that consider either the XCI process or its escape (XCE). To take into account these two underlying biological processes, two genotype coding schemes are commonly used. One corresponds to the assumption of the XCI process as proposed by Clayton (11) while the other corresponds to the XCE process, as implemented in the classical PLINK software (12). For XCI, the proposed coding values are the same for homozygous females and hemizygous males while the heterozygous females fall midway between two homozygous, mimicking the fact that about 50% of cells have the minor (or alternative) allele active while the other 50% of cells have the reference allele active due to random XCI. Using this coding, Clayton derived a one degree-of-freedom score test statistic for case-control studies (11). For XCE, the coding implemented in PLINK codes female genotypes as 0, 1, or 2 copies of the minor allele and male genotypes as 0 or 1 copies of the minor allele. This genotype coding assumes that variants on both copies of the X chromosome are expressed in females. In this XCE setting, Zheng et al. (13) proposed a series of association tests that use different combinations of tests for male and female samples and rely either on genotypic counts or allelic counts in cases and controls. In practice, most of the case-control GWAS investigating X chromosome consider these two coding schemes with a logistic regression model, ignoring the XCI-S process. For time-to-event analysis, the same strategy can be considered by using the classical Cox model but with the same drawback for the XCI-S process. In a recent work, Xu et al. (1) and Han et al. (2) proposed a penalized partial likelihood approach based on the Cox model with a subject-specific random effect that takes into account the XCI-S process. However, the method is quite complex to implement and computationally burdensome for GWAS. We therefore developed a simpler genetic association test accounting for skewed X-inactivation that we used to investigate the role of X-linked genetic variants in the occurrence of ADAs in patients with autoimmune diseases treated by biotherapies.

In this paper, we first present a novel test statistic for selecting interesting loci of the X chromosome in time-to-event data investigation taking into account the XCI-S process that relies on a semi-parametric additive hazard model. It is based on a score-like test evaluated at the null hypothesis that is straightforward to implement. It avoids to compute the complex log-partial likelihood for the random effect Cox model that requires to approximate the integration over the random effects (1, 2). Then, we apply it to the data from the real-world observational multicohort study set-up by the IMI-funded ABIRISK consortium (10) to identify susceptibility loci for drug immunogenicity.

## 2. Materials and Methods

### 2.1. Material

The study population consists of 469 patients with genotyping information from the ABIRISK consortium real-world observational prospective multicenter cohort who suffered from multiple sclerosis, rheumatoid arthritis, and inflammatory bowel diseases and who were treated by biotherapies (10). These patients were naive for the biotherapies they were given during the study, which included tumor necrosis factor (TNF) inhibitors, interferon (IFN)-beta, anti-CD20 (Cluster of Differentiation 20) and anti-interleukin 6 (IL6) receptor monoclonal antibodies.

The patients were followed up for 12 months. Clinical data were recorded in an electronic Case Report Form. DNA samples and serum samples were collected for genetic analyses and ADA testing, respectively. Serum samples for ADA testing were collected at baseline before starting BP and subsequently at each study visit thereafter. Anti-drug antibodies were detected by specific validated assays for each BP and analyzed in central ABIRISK laboratories [more information can be found in Hässler et al. (10)]. The outcome was the time between the date of the first dose of biotherapy and the first detection of the occurrence of anti-drug antibodies. Patients without ADA occurrence were censored at the date of their last clinical visit. Among the 469 subjects, 129 (27.5%) developed ADA during the 1-year follow-up.

The DNA polymorphism analysis was performed with Infinium OmniExpress-24 v1.2 BeadChip. Genotype calling was performed by Genome Studio software 2011.1 with Genotyping module v1.9 (Illumina). Genotypes were called by comparing the generated data with those in the supplied cluster file. The final report for genotype data was based on GRch38/hg38. Quality checks were applied for each sample using autosomal SNPs and removing samples with a call rate (percentage of SNPs genotyped by samples) lower than 95%, excessive observed level of heterozygosity (deviated by more than 3 standard deviations from the mean heterozygosity of the sample), ambiguous sex (genotypic sex different from phenotypic sex from the eCRF), genotyping completeness less than 99%, and non-European ethnicity admixture detected as outliers from a principal component analysis of a linkage-disequilibrium-pruned data set (with a deviation of at least 6 SDs from the mean of at least one of the first 10 principal components). For the quality control specific to the sex chromosomes, we plotted the X chromosome heterozygosity rates and the call rates for SNPs harbored on the Y chromosome. We clustered the individuals using the k-means clustering algorithm and thus eliminated four individuals. As we used the genotyping information and not the information in the measured intensities of X and Y chromosomes, we did not eliminate potential sex-chromosome aneuploidies such as Trisomy X. A total of 457 genotyped individuals were retained.

Then, we extracted 17,565 genotyped SNPs harbored on the X chromosome and conducted further quality control filtering for these SNPs. In practice, we removed samples with a call rate lower than 95% for these SNPs. The SNPs with deviation from Hardy-Weinberg equilibrium test with *p* < 10^−5^ in females, with minor allele frequency less than 5% for both males and females were removed. Finally, a total of 456 genotyped individuals with 12,976 X-chromosome SNPs were considered for subsequent analyses.

### 2.2. Methods

#### 2.2.1. Notation

Let *T* denote the failure time (here the time-to-ADA detection) and *C* the censoring time. We assume that *T* and *C* satisfy the condition of independent and non-informative censoring (14). For each subject *i* (*i* = 1, ..., *n*), *X*_*i*_ = *min*(*T*_*i*_, *C*_*i*_) denotes the observed time of follow-up and δ_*i*_ = **1**_(_*X*__*i*_ = *T*_*i*_)_ the indicator of failure (ADA detection) where the function **1**_(.)_ is the indicator function whose value is 1 if the argument is true and 0 otherwise. We also denote *Y*_*i*_(*t*) = **1**_(*t* ≤ _*X*__*i*_)_ the at-risk process and *N*_*i*_(*t*) = **1**_(_*X*__*i*_ ≤ *t*; δ_*i*_ = 1)_ the counting process, given at time *t*. Let *G* be the genotype for a di-allelic SNP on the X chromosome and denote the two alleles as *A* and *a* with *A* as the rare (or alternative) allele and *a* as the common (or reference) allele. The genotypes are *G* = ([*aa*], [*Aa*], [*AA*]) for female subjects and *G* = ([*a*], [*A*]) for male subjects.

Under the unknown underlying XCI process with a potentially skewed pattern, we consider the following genotype coding variable : (i) females : *W* = (0, 1/2 + *U* × **1**_(*G* = [*Aa*])_, 1) for [*aa*], [*Aa*] and [*AA*], respectively; (ii) males : *W* = (0, 1) for [*a*] and [*A*], respectively. Here, the variable *U* is an unobserved (latent) subject-specific continuous random variable lying in the interval [−1/2, 1/2]. The values of −1/2, 0 and 1/2 represent skewed XCI toward the common allele, random XCI, or skewed XCI toward the rare (or minor) allele, respectively. In the following, we use the rewritten coding : *W* = *Z* + *U* × **1**_(*Z* = 1/2)_ with *Z* = 0, 1/2, 1 for females and *Z* = 0, 1 for males. For each patient *i*, the data consists of (*X*_*i*_, δ_*i*_, *Z*_*i*_, *U*_*i*_).

#### 2.2.2. Survival Model

In this work, we consider a semi-parametric additive hazard model with a latent variable (15, 16). The hazard function for the failure time *T* of individual *i* takes the form:


(1) $$\begin{array}{l} \lambda_{i}\left(t|Z=z,U=u\right)=\lambda_{0}\left(t\right)+\beta z+\beta u\times 1_{\left(z=1/2\right)} \end{array}$$

where β is the unknown regression coefficient of interest, λ_0_(*t*) is an unknown and unspecified baseline hazard function and *U* is the latent (unobserved) variable. Then, the individual-specific (conditional) survival distribution is such that:


$$\begin{array}{l} S_{i}\left(t|Z=z,U=u\right)=exp\left[-\left(\Lambda_{0}\left(t\right)+\beta zt+\beta ut\times 1_{\left(z=1/2\right)}\right)\right]. \end{array}$$

In the following, we assume that the *U*_*i*_ are independent and identically raised cosine distributed random variables with parameters μ = 0 and γ = 1/2 (17). We recall that a continuous random variable *U* is said to have raised cosine distribution with parameters 𝔼(*U*) = μ and γ if its probability density function *f*_*U*_(*x*) is as follows: $f_{U}\left(x\right)=\frac{1}{2\gamma }\left[1+\cos\left(\frac{x-\mu }{\gamma }\pi \right)\right]$ with μ − γ < *x* < μ + γ. In this work, *U* lies in the interval [−1/2, 1/2] and its expectation is equal to zero. Based upon this latter assumption, when marginalized over *U*, the unconditional (or marginal) survival function and hazard function are given by:


$$\begin{array}{l} S\left(t|Z=z\right)=\exp\{-\{\Lambda_{0}\left(t\right)+\beta zt+1_{\left(z=1/2\right)}\\ \;\log\left[\frac{\pi^{2}\sinh\left(1/2\beta t\right)}{1/2\beta t\times \left(\pi^{2}+1/4\beta^{2}t^{2}\right)}\right]\}\} \end{array}$$

$$\begin{array}{l} \lambda \left(t|Z=z\right)=\lambda_{0}\left(t\right)+\beta z+1_{\left(z=1/2\right)}\\ \;\times \left[-1/2\beta \coth\left(1/2\beta t\right)+\frac{1}{t}-\frac{1/2\beta^{2}t}{\left(\pi^{2}+1/4\beta^{2}t^{2}\right)}\right] \end{array}$$

where sinh and coth are the hyperbolic sine function and hyperbolic cotangent function, respectively.

#### 2.2.3. Test Statistic

In this section, a statistic accounting for skewed X-inactivation is derived for testing the null hypothesis *H*_0_:β = 0 (same survival distribution across genotypes) against *H*_1_:β ≠ 0 (different survival distribution across genotypes).

Following Lin and Ying (16), under the marginal additive hazard model introduced just above, a simple semiparametric estimating function for β is constructed and a score-type function is obtained under the null hypothesis (*H*_0_:β = 0). Here, the intensity function for *N*(*t*) is given by:


$$\begin{array}{l} Y\left(t\right)d\Lambda \left(t|Z=z\right)=Y\left(t\right)\{d\Lambda_{0}\left(t\right)+\beta z+1_{\left(z=1/2\right)}\\ \;\times \left[-1/2\beta \coth\left(1/2\beta t\right)+\frac{1}{t}-\frac{1/2\beta^{2}t}{\left(\pi^{2}+1/4\beta^{2}t^{2}\right)}]\right\}. \end{array}$$

By the Doob-Meyer decomposition (14), the counting process *N*(*t*) can be uniquely broken down into the sum of a martingale and a predictable process, such that:


$$\begin{array}{l} N\left(t\right)=M\left(t\right)+\int_{0}^{t}Y\left(t\right)d\Lambda \left(t|Z=z\right). \end{array}$$

Under our model, we can estimate the cumulative hazard function by:


$$\begin{array}{l} {\hat{\Lambda }}_{0}\left(t,\beta \right)=\int_{0}^{t}\frac{\sum_{i=1}^{n}\left\{dN_{i}\left(t\right)-\beta Z_{i}-1_{\left(Z_{i}=1/2\right)}\left[\frac{1/2\beta \cosh\left(-1/2\beta t\right)}{\sinh\left(-1/2\beta t\right)}+\frac{1}{t}-\frac{1/2\beta^{2}t}{\left(\pi^{2}+1/4\beta^{2}t^{2}\right)}\right]\right\}}{\sum_{i=1}^{n}Y_{i}\left(t\right)}. \end{array}$$

Then, following Lin and Ying (16) (Equation 2.7), a simple estimating function (or score-like function) for β can be written as:


$$\begin{array}{l} \;U_{\beta }\left(\beta \right)=\overset{n}{\underset{i=1}{\sum }}\int_{0}^{\infty }\left(Z_{i}\left(t\right)-\overset{-}{Z}\left(t\right)\right)\{dN_{i}\left(t\right)-Y_{i}\left(t\right)\beta Z_{i}dt-\\ Y_{i}\left(t\right)1_{\left(Z_{i}=1/2\right)}\left[\frac{\beta /2\cosh\left(-\beta t/2\right)}{\sinh\left(-\beta t/2\right)}+\frac{1}{t}-\frac{\beta^{2}t/2}{\left(\pi^{2}+\beta^{2}t^{2}/4\right)}\right]dt\} \end{array}$$

with $\overset{-}{Z}\left(t\right)=\frac{\sum_{i=1}^{n}Y_{i}\left(t\right)Z_{i}}{\sum_{i=1}^{n}Y_{i}\left(t\right)}$. Using L'Hopital's rule, we know that the limit of *x* × coth(*x*) as *x* approaches zero is equals to 1. Thus, under *H*_0_:β = 0:


$$\begin{array}{l} U_{\beta }\left(\beta =0\right)=\overset{n}{\underset{i=1}{\sum }}\int_{0}^{\infty }\left(Z_{i}\left(t\right)-\overset{-}{Z}\left(t\right)\right)dN_{i}\left(t\right). \end{array}$$

Under the null hypothesis *H*_0_, the random vector $n^{-1/2}U_{\beta }\left(\beta =0\right)$ converges weakly to a normal with mean zero and with a variance which can be consistently estimated by *n*^−1^*B*(β = 0) with :


$$\begin{array}{l} B\left(\beta =0\right)=\sum \int_{0}^{\infty }{\left(Z_{i}\left(t\right)-\overset{-}{Z}\left(t\right)\right)}^{2}dN_{i}\left(t\right). \end{array}$$

Thus, the (score-like) statistic :


$$\begin{array}{l} S=\frac{U_{\beta }^{2}\left(\beta =0\right)}{B\left(\beta =0\right)} \end{array}$$

is asymptotically distributed under *H*_0_ as a chi-square with one degree of freedom.

A stratified version of the test over *k* strata can be constructed by calculating *U*_β_(β = 0), and its estimated variance, separately in each stratum. Both are then summed over strata. The final stratified test is then calculated in exactly the same way presented just above.

### 2.3. Simulation Study

A simulation study was conducted to assess the size and power of the proposed test (herein called “Score-like”) as compared to three test statistics: (i) the score test of the null hypothesis under the Cox model (herein called “Cox-XCI”) using the XCI coding (*aa* (0), *aA* (0.5) and *AA* (1) for females and *a* (0) and *A* (1) for males); (ii) the score test of the null hypothesis under the Cox model (herein called “Cox-XCE”) using the XCE coding (*aa* (0), *aA* (0.5) and *AA* (1) for females and *a* (0) and *A* (0.5) for males); (iii) the test proposed by Xu et al. (1) and Han et al. (2) based on a random effect XCI-S Cox model (herein called “Xu-Hao”) and implemented in the R package “xlink” (18). To be in line with the Xu et al.'s XCI-S test that takes into account the sex as a potential confounding factor, we compared the results obtained by the Xu-Hao test to those obtained with the stratified versions (by the sex) of the Score-like, Cox-XCI and Cox-XCE test statistics.

Data were simulated under various scenarios assuming a locus undergoing XCI-S. The simulated variables were sex (females *K* = 2 and males *K* = 1), SNP genotype (females: *Z* = 0 for *aa*, *Z* = 1/2 for *Aa* and *Z* = 0 for *AA*; males: *Z* = 0 for *a* or *Z* = 1 for *A*), the individual skewness parameter and the time-to-event. In practice, genotype information for females was generated by combining the values of two Bernoulli variables (𝔹(*p*_[*A*]_)) independently drawn and for males from only one Bernoulli variable with mean: 10%, 20% and 30% for both males and females. This value corresponds to a pseudo-minor allele frequency (MAF), i.e., the proportion of [*A*] allele in the simulated population. The ratio between the female and male rate was set to 1:1. Failure times were generated from an additive hazard model with a protective effect of the minor allele such that the individual-specific hazard rate was: λ(*t*|*Z* = *z, K* = *k*) = λ_0(*k*)_(*t*) + β(*z* + *u* × 1_(*z* = 1/2)_) where λ_0_(*t*) = 5; β = 0, −1.5, −1.75, −2, −2.25, −2.5. The baseline hazard rates were λ_0(*k* = 1)_(*t*) = λ_0_(*t*) for males and λ_0(*k* = 2)_(*t*) = λ_0_(*t*)η for females with η = 1.2. Three distributions for the latent variable *U* were investigated: (i) *U* was generated independently and identically from a raised cosine distribution with parameters μ = 0 and γ = 1/2; (ii) *U* was generated independently and identically from a Beta distribution with 𝔼(*U*) = 1/2 (shape parameters equal to 2); (iii) *U* was generated independently and identically from a truncated normal distribution ranging from −0.5 to 0.5 with mean zero and standard error of 0.18. We investigated no censoring, 20% and 40% type I censoring (administrative censoring). The total number of subjects was chosen to be 400. For each configuration of parameters, 1,000 replications were performed and the levels and powers of the tests were estimated with a 0.05 significance level.

## 3. Results

### 3.1. Simulation Study

As seen from the simulation results, the estimated level of the proposed test under the null hypothesis for a threshold of 0.05 fell within the binomial range [0.0365−0.0635] for all the studied scenarios. This is not the case for the Xu-Hao test, which gave slightly inflated type I error.

For XCI-S with a raised cosine distribution for the skewness parameter (Table 1), the power of the proposed test was always higher than the Xu-Hao test, with a difference varying from 1 to 6% and for each percentage of censoring. Higher power gains where observed for larger MAF. As expected, the Xu-Hao test gave higher power gains than the Cox-XCI test. The Cox-XCE test gave the worst power results. Results observed for XCI-S with a Beta distribution (Table 2) were quite similar. For XCI-S with a truncated Normal distribution (Table 3), for small and moderate MAF (10 and 20%), the power of the proposed test was always higher than the Xu-Hao test, with gains between 1 and 7%. For higher MAF (30%), the power results of the proposed test and the Xu-Hao test were close, sometimes to the slight advantage of the Xu-Hao test.

**Table 1:** Size and power of the tests (Score-like, Cox-XCI, Cox-XCE, Xu-Hao) for XCI-S with raised cosine distribution (threshold level of 0.05).

	**Cens = 0%**					
*p*_*A*_ = 0.10 β:	0	1.5	1.75	2	2.25	2.5
Score-like	4.20	49.10	63.50	75.90	85.90	91.50
Cox-XCI	5.10	40.80	55.60	70.10	81.40	88.20
Cox-XCE	4.70	34.90	48.50	61.90	73.60	82.30
Xu-Hao	7.30	44.30	59.80	72.60	83.00	89.90
*p*_*A*_ = 0.20 β:	0	1.5	1.75	2	2.25	2.5
Score-like	5.10	65.90	80.90	91.40	96.00	98.80
Cox-XCI	5.60	61.50	78.20	89.20	94.70	98.50
Cox-XCE	5.50	53.50	70.50	81.80	92.30	96.80
Xu-Hao	7.20	65.00	79.70	92.40	95.80	98.50
*p*_*A*_ = 0.30 β:	0	1.5	1.75	2	2.25	2.5
Score-like	4.60	75.70	91.30	95.80	99.00	99.80
Cox-XCI	4.60	72.80	89.10	94.10	98.40	99.80
Cox-XCE	5.70	63.90	83.30	90.60	97.20	99.30
Xu-Hao	7.10	75.10	90.30	95.20	98.70	99.80
	**Cens = 20%**					
*p*_*A*_ = 0.10 β:	0	1.5	1.75	2	2.25	2.5
Score-like	6.10	42.00	52.20	64.60	76.10	84.10
Cox-XCI	5.60	32.00	43.60	55.80	67.40	76.70
Cox-XCE	6.20	27.90	37.00	48.90	59.40	69.00
Xu-Hao	8.00	36.20	47.30	59.80	70.50	80.90
*p*_*A*_ = 0.20 β:	0	1.5	1.75	2	2.25	2.5
Score-like	5.80	55.50	73.40	81.50	91.90	97.00
Cox-XCI	5.90	49.10	67.60	76.90	89.40	96.40
Cox-XCE	6.30	43.50	57.70	70.50	83.10	91.80
Xu-Hao	7.80	53.20	71.80	80.20	91.40	96.70
*p*_*A*_ = 0.30 β:	0	1.5	1.75	2	2.25	2.5
Score-like	4.30	67.10	81.40	92.80	97.80	99.70
Cox-XCI	4.80	61.80	77.40	90.30	97.00	99.30
Cox-XCE	4.50	54.30	69.40	85.10	93.30	97.20
Xu-Hao	6.90	64.70	79.60	91.30	97.50	99.40
	**Cens = 40%**					
*p*_*A*_ = 0.10 β:	0	1.5	1.75	2	2.25	2.5
Score-like	5.70	34.90	41.70	53.40	63.00	75.00
Cox-XCI	5.10	24.60	29.70	41.80	50.20	64.00
Cox-XCE	5.80	20.60	26.20	35.80	42.40	54.30
Xu-Hao	7.20	28.10	35.00	46.30	54.90	68.00
*p*_*A*_ = 0.20 β:	0	1.5	1.75	2	2.25	2.5
Score-like	5.20	46.10	58.00	72.80	79.00	92.20
Cox-XCI	5.00	38.80	51.40	66.30	74.40	89.10
Cox-XCE	5.90	33.10	43.60	57.40	67.30	80.50
Xu-Hao	6.40	43.30	55.40	69.70	77.80	91.30
*p*_*A*_ = 0.30 β:	0	1.5	1.75	2	2.25	2.5
Score-like	5.00	57.60	73.90	81.50	92.60	97.40
Cox-XCI	5.30	52.50	69.10	78.60	90.80	96.20
Cox-XCE	4.80	45.70	57.60	71.00	84.30	92.40
Xu-Hao	7.40	56.70	72.20	81.50	92.50	96.90

**Table 2:** Size and power of tests (Score-like, Cox-XCI, Cox-XCE, Xu-Hao) for XCI-S with Beta distribution (threshold level of 0.05).

	**Cens = 0%**					
*p*_*A*_ = 0.10 β:	0	1.5	1.75	2	2.25	2.5
Score-like	5.10	60.70	78.00	85.40	94.50	98.30
Cox-XCI	5.20	54.90	72.60	82.00	92.60	97.30
Cox-XCE	4.40	54.80	73.30	82.50	93.30	97.10
Xu-Hao	7.30	50.50	69.80	78.30	89.10	96.10
*p*_*A*_ = 0.20 β:	0	1.5	1.75	2	2.25	2.5
Score-like	4.20	78.90	91.00	97.70	99.50	100.00
Cox-XCI	4.80	76.00	89.70	96.90	99.20	100.00
Cox-XCE	4.60	76.60	89.40	96.70	99.20	99.90
Xu-Hao	6.40	72.40	87.10	94.90	98.90	99.90
*p*_*A*_ = 0.30 β:	0	1.5	1.75	2	2.25	2.5
Score-like	5.20	82.00	94.00	98.80	99.80	100.00
Cox-XCI	5.70	80.30	93.20	98.80	99.80	100.00
Cox-XCE	5.90	78.80	93.20	98.00	99.70	100.00
Xu-Hao	7.90	79.50	91.60	98.10	99.50	100.00
	**Cens = 20%**					
*p*_*A*_ = 0.10 β:	0	1.5	1.75	2	2.25	2.5
Score-like	5.00	52.80	67.00	83.00	89.00	95.10
Cox-XCI	5.30	44.20	59.90	77.40	84.80	92.50
Cox-XCE	4.40	44.20	60.10	75.50	83.40	92.60
Xu-Hao	7.60	40.70	55.70	72.80	83.30	88.90
*p*_*A*_ = 0.20 β:	0	1.5	1.75	2	2.25	2.5
Score-like	5.10	69.90	85.50	92.60	97.90	99.80
Cox-XCI	5.10	65.50	81.40	91.40	97.60	99.70
Cox-XCE	5.90	64.90	82.80	90.70	97.20	99.30
Xu-Hao	7.10	62.80	78.00	88.90	95.70	99.10
*p*_*A*_ = 0.30 β:	0	1.5	1.75	2	2.25	2.5
Score-like	4.50	78.20	88.50	97.30	99.60	100.00
Cox-XCI	5.20	76.60	88.00	96.70	99.50	100.00
Cox-XCE	4.70	73.70	86.50	96.10	99.20	100.00
Xu-Hao	8.20	76.10	87.40	94.20	99.20	99.70
	**Cens = 40%**					
*p*_*A*_ = 0.10 β:	0	1.5	1.75	2	2.25	2.5
Score-like	4.60	45.30	56.50	69.10	81.10	89.30
Cox-XCI	4.80	35.40	45.60	59.20	73.60	84.00
Cox-XCE	5.50	34.70	47.00	57.40	74.50	82.20
Xu-Hao	6.50	34.00	44.80	56.10	68.00	79.00
*p*_*A*_ = 0.20 β:	0	1.5	1.75	2	2.25	2.5
Score-like	4.40	59.70	74.90	85.00	93.70	98.50
Cox-XCI	4.60	54.00	68.40	82.50	92.30	97.20
Cox-XCE	4.60	54.10	67.90	82.20	92.30	97.80
Xu-Hao	6.60	51.80	66.70	78.80	88.60	95.80
*p*_*A*_ = 0.30 β:	0	1.5	1.75	2	2.25	2.5
Score-like	5.80	64.80	78.30	90.20	96.80	99.10
Cox-XCI	5.70	61.90	76.60	89.20	96.40	98.90
Cox-XCE	6.30	60.10	74.40	87.50	96.00	98.70
Xu-Hao	9.20	62.30	76.60	86.70	95.90	98.00

**Table 3:** Size and power of tests (Score-like, Cox-XCI, Cox-XCE, Xu-Hao) for XCI-S with truncated normal distribution (threshold level of 0.05).

	**Cens = 0%**					
*p*_*A*_ = 0.10 β:	0	1.5	1.75	2	2.25	2.5
Score-like	4.00	47.20	61.20	75.10	85.50	93.30
Cox-XCI	5.30	40.80	53.50	69.60	80.80	89.90
Cox-XCE	5.00	34.60	46.90	62.00	73.70	84.90
Xu-Hao	7.80	43.70	57.40	71.80	83.80	91.50
*p*_*A*_ = 0.20 β:	0	1.5	1.75	2	2.25	2.5
Score-like	5.10	65.70	81.40	90.40	96.20	98.80
Cox-XCI	5.40	61.00	77.90	87.90	95.50	98.60
Cox-XCE	5.60	54.50	70.90	81.20	91.30	96.60
Xu-Hao	7.90	65.40	79.90	90.30	96.20	98.80
*p*_*A*_ = 0.30 β:	0	1.5	1.75	2	2.25	2.5
Score-like	5.40	77.00	88.30	94.10	99.20	100.00
Cox-XCI	5.70	74.50	87.40	93.60	99.00	99.90
Cox-XCE	6.00	63.90	82.50	90.30	97.00	99.10
Xu-Hao	8.90	78.60	89.20	94.60	99.20	99.80
	**cens = 20%**					
*p*_*A*_ = 0.10 β:	0	1.5	1.75	2	2.25	2.5
Score-like	5.10	41.20	52.30	65.60	76.80	84.20
Cox-XCI	5.10	31.20	42.40	56.60	69.00	77.90
Cox-XCE	5.00	26.40	36.00	49.10	60.40	70.60
Xu-Hao	8.30	34.70	47.00	61.20	72.10	79.40
*p*_*A*_ = 0.20 β:	0	1.5	1.75	2	2.25	2.5
Score-like	4.70	56.90	69.90	84.50	92.30	97.10
Cox-XCI	4.20	50.30	66.30	81.10	90.40	96.00
Cox-XCE	4.50	44.60	58.20	74.10	84.00	92.70
Xu-Hao	6.20	55.30	69.50	83.60	91.00	97.10
*p*_*A*_ = 0.30 β:	0	1.5	1.75	2	2.25	2.5
Score-like	5.00	68.80	81.10	92.30	97.40	98.90
Cox-XCI	4.50	65.10	78.80	90.70	96.80	98.50
Cox-XCE	5.40	55.60	72.00	84.20	93.20	96.90
Xu-Hao	7.10	69.90	82.40	93.60	97.30	98.80
	**Cens = 40%**					
*p*_*A*_ = 0.10 β:	0	1.5	1.75	2	2.25	2.5
Score-like	6.20	33.10	44.20	55.40	64.00	74.20
Cox-XCI	5.40	24.00	33.10	43.90	52.10	64.20
Cox-XCE	6.10	19.00	28.90	37.00	43.60	55.40
Xu-Hao	8.10	27.30	36.80	45.70	54.70	68.50
*p*_*A*_ = 0.20 β:	0	1.5	1.75	2	2.25	2.5
Score-like	5.20	45.00	57.90	73.20	81.60	90.80
Cox-XCI	4.80	39.30	50.90	67.50	76.50	87.60
Cox-XCE	4.50	32.50	43.70	58.90	68.80	80.70
Xu-Hao	7.10	42.80	55.20	72.30	80.10	89.70
*p*_*A*_ = 0.30 β:	0	1.5	1.75	2	2.25	2.5
Score-like	4.70	56.20	70.10	84.10	91.70	96.80
Cox-XCI	4.40	51.80	66.50	80.90	89.80	96.40
Cox-XCE	4.70	43.80	57.30	73.70	83.70	92.10
Xu-Hao	8.10	58.20	71.50	84.60	91.30	96.80

### 3.2. Abirisk Cohort

The cohort analyzed in this work consists in 456 patients with genotyping information who successfully passed the quality-control procedures and who were suffering from auto-immune diseases and were naive for the studied biotherapies before the study. There were 309 women (68%) and 147 men (32%). Patients were aged from 18 to 87 years old and the median age was 41 years old. In this multi-cohort, 131 patients (29%, 65 males, 66 females) suffered from inflammatory bowel diseases (Crohn's disease or ulcerative colitis), 141 (31%, 42 males, 99 females) from multiple sclerosis and 184 (40%, 40 males, 144 females) from rheumatoid arthritis. Eight biotherapies were used in the study : TNF-inhibitors (Adalimumab, Etanercept, Infliximab), IFNβ (IFNβ-1a subcutaneous, IFNβ-1a intra-muscular and IFNβ-1b subcutaneous), anti-IL6R (Tocilizumab) and anti-CD20 monoclonal antibodies (Rituximab). 253 patients (55%) were taking TNF-inhibitors, 141 (31%) IFNβ, 35 (8%) anti-IL6R and 27 (6%) anti-CD20. For the 456 patients, the probability of producing ADA at 1 year was 27.5% [23.0%-31.8%]. The sex variable was not significantly related to the time to ADA detection (*p* = 0.64).

We first computed the *p*-values obtained with the stratified version (by the sex) of the score-like test. Then, to identify X-chromosomal loci associated with ADAs, we performed an FDR-based genome-wide analysis. Controlling the FDR at nominal level of 5% (19), we selected 24 associated signals. Results obtained using unstratified tests were similar. Among these association signals, two signals had *p*-values lower than 10^−6^ : rs5991366 (*p* = 3.56*10^−8^ stratified test, *p* = 4.58*10^−8^ unstratified test) and rs5991394 (*p* = 3.74*10^−7^ stratified test, *p* = 3.63*10^−7^ unstratified test). Both SNPs were located in the cytoband Xp22.2 near the gene chromobox 1 pseudogene 4 (*CBX1P4*) and the gene *REPS2* (*RALBP1* Associated Eps Domain Containing 2). For rs5991366, the frequency for the minor allele was 9.1% for females and 8.8% for males with no significant difference. For rs5991394, the frequency for the minor allele was 9.9% for females and 10.2% for males with no significant difference. This pair of SNPs are in very high linkage disequilibrium (*R*^2^ = 0.85) (20).

**Figure 1:**
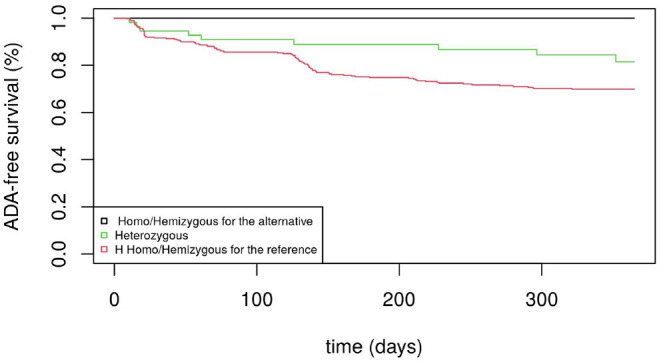
ADA-free survival curves (men and women) for rs5991366.

**Figure 2:**
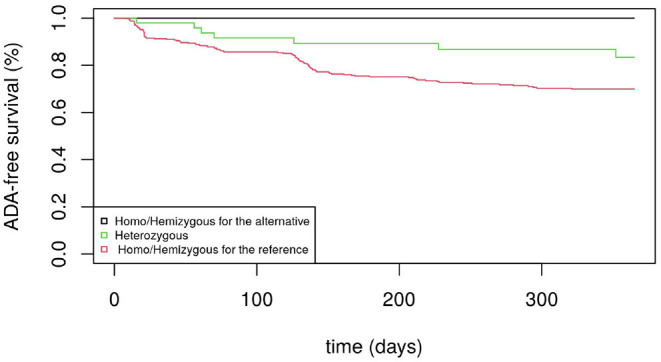
ADA-free survival curves (men and women) for rs5991394.

Figure 1 displays the Kaplan-Meier survival curves for the SNP rs5991366. No event occurred among the 13 hemizygous males and 4 homozygous females for the alternative allele, whereas among the hemizygous males and homozygous females for the reference allele, 26.1% (35/134) and 27.6% (71/257) developed ADA positivity, respectively. Among the heterozygous females, 14.6% (7/48) developed ADA positivity. Figure 2 displays the Kaplan-Meier survival curves for the SNP rs5991394. No event occurred among the 15 hemizygous males and 3 homozygous females for the alternative allele, whereas among the hemizygous males and homozygous females for the reference allele, 26.5% (35/132) and 27.5% (69/251) developed ADA positivity, respectively. Among the heterozygous females, 16.4% (9/55) developed ADA positivity.

**Figure 3:**
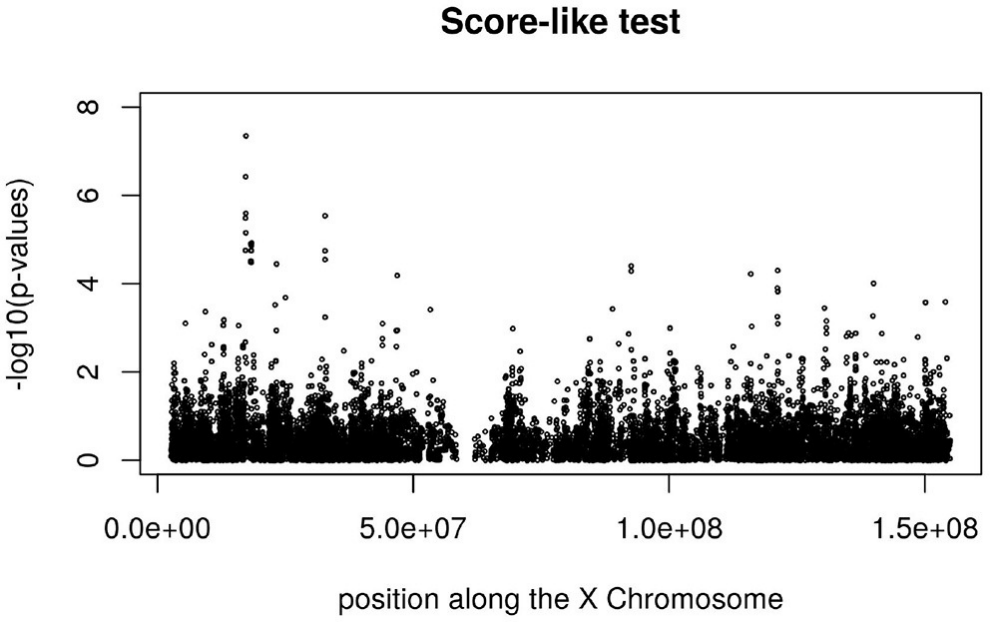
Manhattan plot of X Chromosome genome-wide association results-Score-like test.

**Figure 4:**
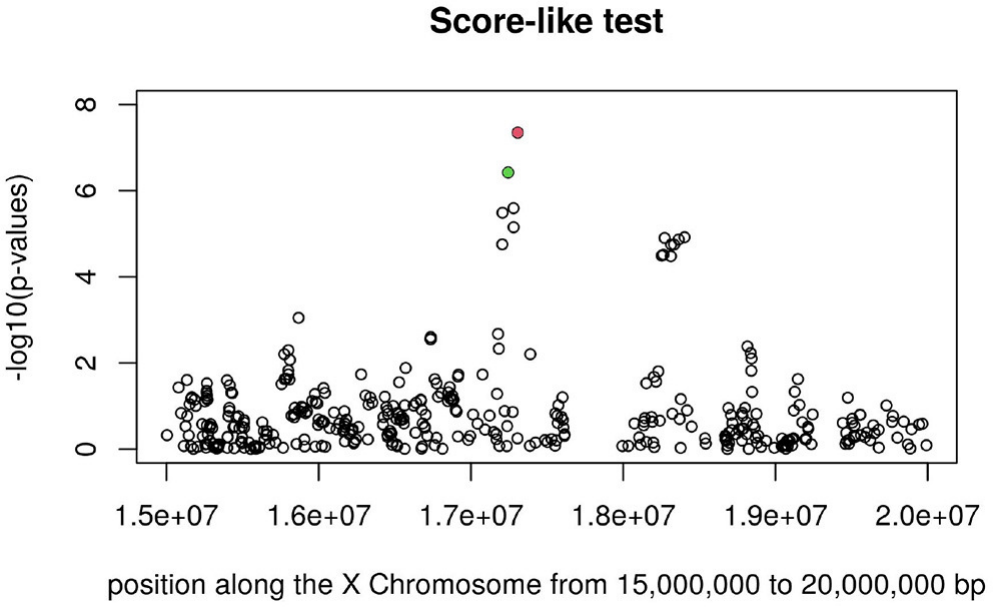
Manhattan plot of X Chromosome genome-wide association results (Score-like test) with zoom in on area 15,000,000–20,000,000 with SNP rs5991366 (red) and SNP rs5991394 (green).

**Figure 5:**
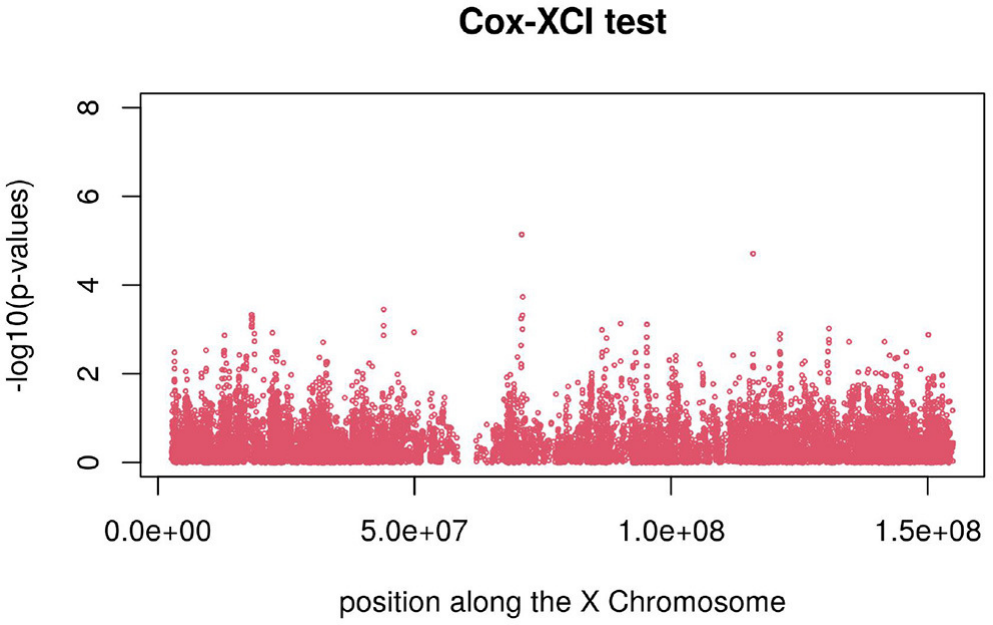
Manhattan plot of X Chromosome genome-wide association results-Cox-XCI test.

**Figure 6:**
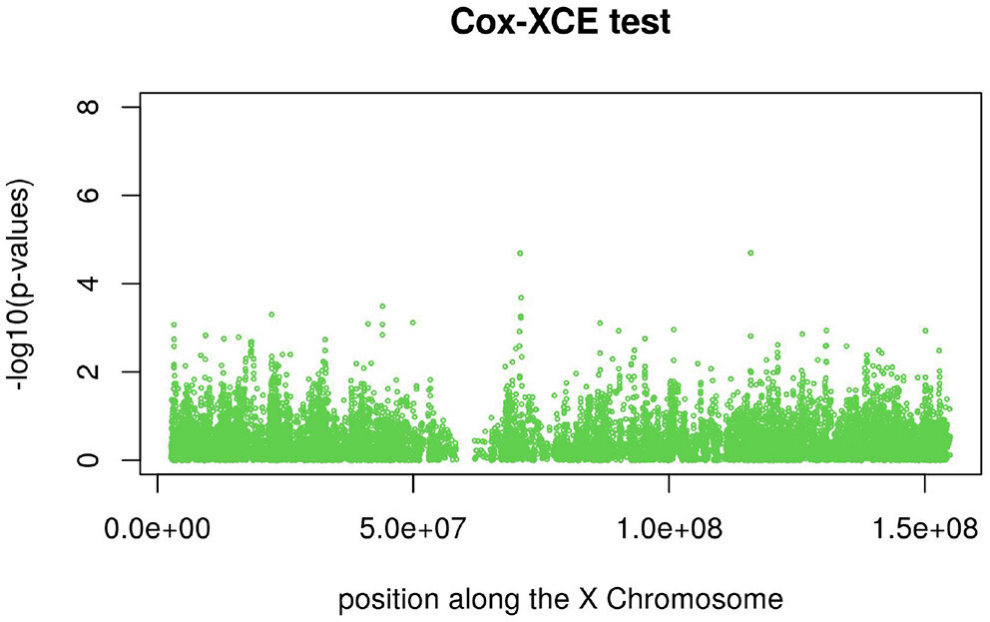
Manhattan plot of X Chromosome genome-wide association results-Cox-XCE test.

**Figure 7:**
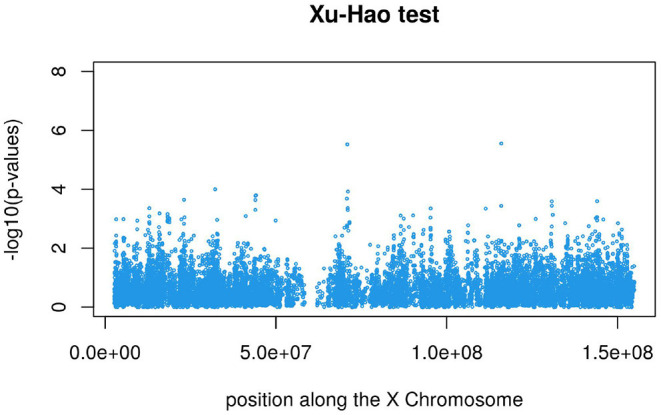
Manhattan plot of X Chromosome genome-wide association results - Xu-Hao test.

Figures 3, 4 display the Manhattan plot of the X Chromosome genome-wide association results obtained with the score-like test (Figure 3) with a zoom in on the genomic region 150,000,000 bp to 20,000,000 bp (Figure 4). Figures 5–7 display Manhattan plots of the X Chromosome genome-wide association results obtained with the (stratified) Cox-XCI test (Figure 5), the (stratified) Cox-XCE test (Figure 6) and the Xu-Hao test (Figure 7). Looking at the Manhattan diagrams in the distal Xp22.2 region, the association signals obtained from the Cox-XCI, Cox-XCE and Xu-Hao tests were weaker than those obtained from the score-like test.

## 4. Discussion

Our aim was to investigate the association of loci located on the X chromosome with drug immunogenicity in auto-immune diseases, while taking into account different X-chromosome inactivation models: XCI (random inactivation), XCI-S (skewed inactivation) and XCE (inactivation escape). To date, few strategies have been proposed for analyzing time-to-event data, taking into account the complexity of the X-chromosome inactivation biological process. In practice, one can use statistical tests derived from the classical Cox model (with XCI or XCE coding) or a more complex and computationally burdensome test based on a random effect Cox model (1, 2).

We propose a new score-like test that takes into account for an unknown underlying XCI process with a potentially skewed pattern. We assumed a semi-parametric additive hazard model with a latent factor that provides an easy and meaningful interpretation of the skewed X-inactivation process. Results from the simulation study show that the proposed test provides higher power gains than the score tests from the Cox model (XCI and XCE coding) and to the likelihood ratio test proposed by Xu et al. (1) and Han et al. (2). With the latter test, some caution should be taken when interpreting its power results as the type I error rate is slightly inflated. For the distribution of the latent factor, we considered a raised cosine distribution that mimicks the unknown skewed X-inactivation process and leads to a closed form for the marginal survival distribution. Other choices are possible. However, as shown by the simulation study, the proposed test performs quite well even with other distributions such as the Beta and truncated Normal distributions. In the present, the additive hazard model that represents the effect of the genetic locus on the hazard rate as a linear form, serves as an alternative to the usual proportional hazards model and benefits from several useful mathematical properties. The model involves a straightforward simple testing procedure and a stratified version of the score-like test is easily obtained. However, if the genetic effect is not confounded with sex, there is no need to stratify by sex in the analysis. Note that our main objective was not to find the best genetic model over various biological processes but to propose a novel statistic for testing the effect of a loci under an XCI-S process. The statistic could nevertheless be used for model selection, although this would require further works.

By investigating the association between the genes located on the X chromosome with anti-drug immunogenicity, the proposed test allowed us to select two SNPs with high linkage disequilibrium (rs5991366 and rs5991394) located in the cytoband Xp22.2 that would have been overlooked by the score tests from the Cox model (XCI and XCE coding) and the Xu et al.'s likelihood ratio test. Both SNPs show a similar protective effect for drug immunogenicity without any occurrence of ADA positivity for homozygous females and hemizygous males for the alternative allele. In contrast, almost 30% of the homozygous females and hemizygous males for the reference allele experienced ADA positivity. Note that for both SNPs, the frequencies of the alternative allele observed for both males and females were not significantly different from the estimates obtained in European samples (21).

The region containing the two X-linked SNPs associated with ADA occurrence is conserved between primates (99% identity) and also within mammals (70% identity with mus musculus) (22). Moreover, the SNP rs59913394 is in the proximity of a regulatory variant (rs113772781) in LD (r2=1 in Europeans), but no gene expression in immune cell types is regulated by this variant (23). In the genomic neighborhood of these two SNPs, the closest gene, *CBX1P4* (Chromobox 1 Pseudogène 4), is a pseudogene followed by the gene *REPS2* (*RALBP1* Associated Eps Domain Containing 2). The *REPS2* gene (*RALBP1* Associated Eps Domain Containing) encodes for a protein which is part of a protein complex that regulates the internalization of growth factors receptors such as *EGF* and insulin receptors and may have an inhibitory effect on growth factor cell signaling. It is downregulated in prostate cancer progression and that this downregulation is accompanied by upregulation of *NF-*κ*B* activity (24, 25). No direct effect of *REPS2* on auto-immune disease has been recognized to date. However, the *REPS2* gene is widely expressed in several human tissues, including white blood cells and lymph nodes. Its biological targets (growth factors and *NF-*κ*B* signaling) are also widely expressed and have a major role in inflammation and immunity. Dysregulation of the IGFs pathway has an important role in autoimmune diseases (26). In particular, IGF stimulates Treg proliferation and has a protective effect in autoimmune disease models. Further in the Xp22.2 locus, several genes have a role in immunity including *ACE2, TLR7*, and *TLR8*. *ACE2* (Angiotensin I converting enzyme 2) is a key actor of the renin-angiotensin system acting as a homeostatic regulator of the vascular function (blood pressure regulation). Recently it attracted much attention for its major role in SARS-CoV-2 infection through its affinity for the viral spike factor, raising the question of a possible sex bias in this disease (27). The genes *TRL7* and *TLR8* are members of the Toll-like receptor (TLR) family which plays a major role in activation of innate immunity. Studies have shown that some immunity genes such as *TLR7, CD40LG*, and *CXCR3* may escape XCI in several lymphoid cells (B cells, T cells, plasmacytoid cells), especially in some auto-immune diseases such as Systemic Lupus Erythematosus due to a dysregulation of the XIST long non-coding RNA involved in XCI (28–30). Here, we investigated the genes that are located in the vicinity of the SNPs of interest, but other genes that are further from the SNPs (e.g., *MNG2* multinodular goiter 2) could be relevant. Additional studies are required to strengthen our findings.

In the search for common susceptible loci, we analyzed together the time to ADA of patients treated with eight different drugs for different autoimmune diseases. This strategy, which uses information concerning various therapies and autoimmune diseases, is likely to provide significant gain in power in finding loci not associated with specific therapies or autoimmune diseases. Nevertheless, it would not be suitable for searching for loci that are specific to a particular therapy or disease.

In conclusion, we think that more X-Chromosome GWAS should be performed and that the proposed test, which is easy to implement with standard softwares, is well-suited for identifying X Chromosome SNPs, while taking into account all possibilities of the skewed X-Chromosome inactivation process.

## Data Availability Statement

The data analyzed in this study were collected in the context of the ABIRISK project by ABIRISK partners. Access to the minimal dataset underlying the findings can be obtained upon request to the ABIRISK Sustainability Scientific Committee by submission of an analysis plan. The analysis plan should explain the purpose of the use of the data and confirm the intention to use the data only for replication studies concerning anti-drug inhibitors, since this is the limitation of the ethical permission on how this data can be used. The contact person of the ABIRISK Sustainability Scientific Committee to whom the requests should be sent is MP (marc.pallardy@inserm.fr).

## Ethics Statement

The studies involving human participants were reviewed and approved by Medical Ethics Committee of the General University Hospital in Prague (reference 125/12, Evropský grant 1.LF UK-CAGEKID) Institutional Committee of Heinrich Heine University, Düsseldorf, Germany (protocol reference 4451) Ethikkommission der Fakultät für Medizin der Technischen Universität München, München, Germany (reference 335/13) Ethikkommission Nordwest- und Zentralschweiz, Basel (reference 305/13) Ethikkommission der Medizinischen Universität Inns- bruck, Innsbruck (reference AN2013-0040 331/2.1) Comité Ético de Investigación Clínica de l'Hospital Universitari Vall d'Hebrón, Barcelona [reference EPA(AG)66/2013(3866)] Stockholm Regional Ethics Committee, Stockholm (reference Dnr. 2013/1034-31/3 and Dnr. 2015/749-32) Comité de Protection des Personnes Ile de France VII (reference 13-048) Medical Ethical Committee of the Academisch Medisch Centrum, Amsterdam (reference 2013-304#B20131074) Local Ethics Committee of AOU Careggi (reference Protocol No 2012/0035982) NRES Committee London, City and East (reference 14/LO/0506) Comité de Protection des Personnes Ile de France IV (reference 2013/24) Comité d'éthique hospitalo-facultaire universitaire de Liège (reference 2015/55). The patients/participants provided their written informed consent to participate in this study.

## Author Contributions

PB coordinated the project and developed the proposed statistical procedure. SH, SC-B, and PB participated in writing the original draft. SH and PB analyzed the data. SH, MA, FD, AF-H, XM, MP, and PB participated in the data collection. MP coordinated the ABIRISK project. All authors read and approved the final manuscript.

## Conflict of Interest

The authors declare that the research was conducted in the absence of any commercial or financial relationships that could be construed as a potential conflict of interest. The handling editor EJ declared a past co-authorship with the author SH.

## Publisher's Note

All claims expressed in this article are solely those of the authors and do not necessarily represent those of their affiliated organizations, or those of the publisher, the editors and the reviewers. Any product that may be evaluated in this article, or claim that may be made by its manufacturer, is not guaranteed or endorsed by the publisher.
